# Patients’ expectations surrounding revision total hip arthroplasty: a literature review

**DOI:** 10.1186/s42836-024-00250-6

**Published:** 2024-06-03

**Authors:** Omar Mohammad, Shahril Shaarani, Adnan Mohammad, Sujith Konan

**Affiliations:** 1 King’s College Hospital, Denmark Hill, London, SE5 9RS UK; 2 Department of Trauma & Orthopaedics, University College London Hospitals, Ground Floor, 250 Euston Road, London, NW1 2PG UK; 3 East Surrey Hospital, Canada Avenue, Redhill, RH1 5RH UK

**Keywords:** Expectation, PROM, Satisfaction, THA, Revision total hip arthroplasty

## Abstract

**Background:**

Revision total hip arthroplasties (RTHA) are associated with a higher complication rate than primary total hip arthroplasties (THA), and therefore it is important for patients to have realistic expectations regarding outcomes. The aim of this literature review was to gather and summarize the available evidence on patients’ expectations following RTHA.

**Methods:**

A literature search was conducted in PubMed, PsycINFO, Cochrane, Google Scholar, Web of Science and Embase from inception to November 2023. Articles assessing patient expectations for RTHA were included. Methodological quality was assessed by two independent reviewers using the National Heart, Lung and Blood Institute (NIH) study quality assessment tool for observational cohort and cross-sectional studies. A qualitative analysis was performed involving the summarization of study characteristics and outcomes.

**Results:**

The search strategy generated 7,450 references, of which 5 articles met the inclusion criteria. Methodological quality scores ranged from 7–10. Patients had high expectations concerning future walking ability, pain and implant longevity relative to actual postoperative outcomes. A significant positive correlation was found between fulfilled expectations of pain and walking ability and patient satisfaction (*r* = 0.46–0.47). Only two studies assessed the fulfillment of patient expectations. Great variability was seen in the measurement of expectations.

**Conclusion:**

Patients undergoing RTHA appeared to have high expectations for pain and functionality compared to postoperative outcomes. However, there was a paucity of high-quality data in this area, limiting the accuracy of the conclusion. Further research is needed, that emphasizes developing a sound theoretical framework for expectations, allowing for the consistent implementation of valid measurement tools for patient expectations.

## Introduction

Total hip arthroplasty (THA) is a cost-effective procedure for improving a patient’s quality of life (QOL), pain, and function when conservative therapies have failed [1–3]. Despite the widely recognized success of THA, there is a certain level of risk that may necessitate a revision procedure. The incidence of revisions is on the rise and is projected to increase by 31% by 2030 in England & Wales, UK [4].

When compared to primary THA, revision THA (RTHA) is associated with higher rates of short- and long-term complications, elevated mortality rates, lower satisfaction, and smaller improvements in functional outcomes [5–11]. Whether patients undergo either primary or RTHA, they largely expect a reduction in pain and an improvement in both function and quality of life [12–15]. In the preoperative period, it is important to assess these expectations, to ensure that patients have a realistic perspective of the outcomes of the operation and are not dissatisfied. Aside from technical factors and the quality of existing bone, patient factors may also partially explain the less favourable outcomes of RTHA relative to primary THA [8].

There is a growing body of literature across a variety of medical specialties linking clinical outcomes with patients’ expectations and satisfaction. Patient satisfaction has been shown to lead to higher compliance and attendance for monitoring and follow-up care [16], which are integral factors in optimizing prosthesis longevity. Furthermore, patients’ expectations are strongly correlated with satisfaction, with satisfied patients having their expectations fulfilled [17] and unrealistic expectations being correlated with dissatisfaction [18]. This has led to increasing emphasis on measures of quality of life and patients’ feelings of satisfaction [19, 20]. Therefore, as a reflection of this shift in emphasis, it has become essential to gain a better understanding of patients’ expectations.

Although patient expectations have been widely discussed in current primary THA research [17, 18, 21], there is an apparent sparsity in the RTHA literature. This literature review therefore aimed to comprehensively assess all relevant studies evaluating the expectations of patients undergoing RTHA, and how this in turn relates to post-operative outcomes where possible.

## Materials and methods

### Search strategy

A comprehensive electronic literature search was performed in the following databases: PubMed, The Cochrane Library, Google Scholar, PsychINFO, Web of Science and Embase to identify eligible studies published until the 7th November 2023. Search terms were derived from MeSh terms in PubMed and free text terms relating to (1) hip arthroplasty, (2) revision and (3) expectations/expectancies (Table 1). Although Haanstra et al. offered distinct definitions for expectations and expectancies as being “cognitions regarding probable future events” and “the act or state of expecting” [22], the current literature uses the two terms interchangeably to show that an individual is “expecting something to occur in the future”. Therefore, whilst they are different concepts, no distinction was acknowledged between the two.

**Table 1:** Search terms used for each database (date of search: 7th November 2023)

Database	Search terms
PubMed	“Tha” OR “thr” OR “total hip arthroplasty” OR “hip replacement” OR “hip prosthesis” OR “joint prosthesis” OR “joint replacement” OR “arthroplasty, replacement, hip” OR ((“Arthroplasty” OR “arthroplasty, Replacement”) AND (“hip” OR “hip joint”)) OR “Osteoarthritis, Hip/surgery” OR “Osteoarthritis, Hip/therapy” OR “hip osteoarthritis” AND (“Expectations” OR “expectancies” OR “Postoperative expectations” OR “Preoperative expectations” OR “Self-Efficacy” OR “Health Knowledge, Attitudes, Practice” OR “expectancy” OR “expectance” OR “credibility” OR “patient preference” OR “Satisfaction” OR “satisfied”) AND (“Revision” OR “reoperation” OR “secondary”)
Cochrane library	#1 “THA”:ti,ab,kw or “total hip arthroplasty”:ti,ab,kw #2 hip prosthesis:ti,ab,kw or hip replacement:ti,ab,kw #4 expectations:ti,ab,kw or expectancies:ti,ab,kw or satisfaction:ti,ab,kw or satisfied:ti,ab,kw #5 revision:ti,ab,kw or “revision surgery”:ti,ab,kw #6: #1 or #2 and #4 and #5
Google scholar	Expectations OR satisfaction AND THA OR “total hip arthroplasty” OR “joint prosthesis” OR “hip prosthesis” OR “hip replacement” OR “joint replacement” AND revision
Web of Science	#1 TS = (Revision) OR TS = (Re operation) OR TS = (revisional) OR TS = (satisfaction) #2 TS = (expectations) OR TS = (expectancies) OR TS = (preoperative expectations) OR TS = (post-operative expectations) #3 TS = (total hip arthroplasty) OR TS = (tha) OR TS = (hip replacement) OR TS = (thr) OR TS = (joint replacement) OR TS = (joint prosthesis) OR TS = (hip prosthesis) #4: #1 and #2 and #3
PsycInfo	(hip replacement OR tha OR total hip arthroplasty OR joint replacement OR thr OR hip prosthesis OR joint prosthesis) AND (expectancies OR expectations OR post-operative expectations OR pre-operative expectations OR satisfaction OR satisfied) AND (Revision OR revisional)
Embase	#1 (Tha or thr or total hip arthroplasty or hip replacement or hip prosthesis or joint prosthesis or joint replacement or Arthroplasty).mp. #2 (Revision or reoperation or secondary).mp. #3 (Expectation$ or expectancies or Postoperative expectations or Preoperative expectations or Self-Efficacy or expectancy or expectance or credibility or patient preference or Satisfaction or satisfied).mp. #4: #1 and #2 and #3

### Inclusion criteria

The individual search results from each database were combined barring duplicates, and the remaining titles and abstracts were then screened against the inclusion criteria found below.

The studies had to meet the following inclusion criteria to be eligible:

1. The study included revision THA patients;
2. Patients’ expectations were assessed;
3. The study had to be written in English;
4. The patients were adults > 18 years of age.

If an article assessed both primary and RTHA groups but failed to report the data separately for each group, the study was excluded, as we would not be able to extract the relevant data.

Two reviewers (OM and SRS) independently assessed the full text articles, based on the title and abstract, against the inclusion criteria. If there was any uncertainty regarding the eligibility of a study the full text was examined. The results of the search are shown in Table 2.

**Table 2:** Scores on the methodological quality assessment. Quality was rated as poor (0–4 out of 14 questions), fair (5–10 out of 14 questions), or good (11–14 out of 14 questions); NA: not applicable, NR: not reported, CD: can’t determine

**Reference**	Eisler et al	Haddad et al	Barrack et al	Hellman et al	Zhang et al
**Journal**	*J. Arthro*	*J. Arthro*	*CORR*	*Iowa Orth. J*	*J. Orth*
**Year**	2002	2001	2006	1996	2023
Was the research question or objective in this paper clearly stated?	√	√	√	√	√
Was the study population clearly specified and defined?	√	√	√	√	√
Was the participation rate of eligible persons at least 50%?	√	√	√	√	√
Were all the subjects selected or recruited from the same or similar populations?	√	√	√	√	√
Was a sample size justification, power description, or variance and effect estimates provided?	X	X	X	√	√
For the analyses in this paper, were the exposure(s) of interest measured prior to the outcome(s) being measured?	√	√	√	X	X
Was the time frame sufficient so that one could reasonably expect to see an association between exposure and outcome if it existed?	√	√	CD	√	√
For exposures that can vary in amount or level, did the study examine different levels of the exposure as related to the outcome?	X	X	X	X	X
Were the exposure measures (independent variables) clearly defined, valid, reliable, and implemented consistently across all study participants?	√	√	√	X	√
Was the exposure(s) assessed more than once over time?	X	X	X	X	√
Were the outcome measures (dependent variables) clearly defined, valid, reliable, and implemented consistently across all study participants?	√	√	CD	√	√
Were the outcome assessors blinded to the exposure status of participants?	X	X	X	X	X
Was loss to follow-up after baseline 20% or less?	√	√	√	√	X
Were key potential confounding variables measured and adjusted statistically for their impact on the relationship between exposure(s) and outcome(s)?	X	√	X	√	√
Summary Quality	Fair 9	Fair 10	Fair 7	Fair 9	Fair 10

### Data extraction and methodological quality assessment

The same two reviewers extracted relevant data from the included studies using a standardized data extraction form (Table 3). The form included information on study design, study population, follow-up period, measurement of expectations and outcome measurements. Moreover, data on the strength of the relationship between expectations and outcomes was extracted where possible (e.g., *P*-values and correlation coefficients).

**Table 3:** Characteristics of included studies

Title	Author, year	Procedure	*n*	Follow-up	Age	Operationalization of expectations	Study design/ measurement level	Expectations	% Patients with fulfilled expectations	Relationship between expectations and satisfaction
Revision total hip arthroplasty: the patient’s perspective	Barrack et al. 2006 [23]	Revision THA	320	At least 1 year after revision THA	N/A	One postoperative question about expectation of revision longevity	Retrospective 3-point Likert scale	Primary THA lasted < 5 yrs: 77% expected revision to last longer; Primary 5–10 yrs: 76% expected revision to last longer; Primary 10–15 yrs: 69% expected the revision to last longer; Primary > 15yrs: 62% expected the revision to last longer	N/A	N/A
Patient expectation and satisfaction in revision total hip arthroplasty	Eisler et al. 2002 [12]	Revision THA	98	1 year	70	a. Two preoperative questions about expectations of future pain and walking ability; b. Postoperatively assessed fulfillment of expectations with two questions	a. Prospective 4-point Likert scale for pre-operative expectations; b. 4-point Likert scale for fulfillment of expectations	92% of patients expected to become pain-free or to have much less pain; 8% expected slightly less pain; 82% expected the same walking ability as after the first THA or very much improved walking ability; 15%, slightly improved; 3%, unchanged walking ability	55%–69%	Fulfilled expectations about pain and walking ability were moderately positively correlated with satisfaction (*r* = 0.46–0.47)
When is total hip arthroplasty a failure? The patients’ perspective	Hellman et al. 1996 [24]	Revision THA	92	At least 2 years after revision THA	66	Two postoperative questions about expectations of revision longevity and how they would feel if they required another revision	Retrospective close-ended multiple-choice questions	35% of patients expected the revision to last for the rest of their lives	N/A	N/A
The expectations of patients undergoing revision hip arthroplasty	Haddad et al. 2001 [14]	Revision THA	60	N/A	70	*Preoperative* assessment using *the Expectation* WOMAC- assessing how they expected to feel in 6 months	Prospective 5-point Likert scale	Expectation WOMAC scores: 7.4 (CI, 6.2–8.6) for pain. Max score: 25; 3.5 (CI, 3.0–4.0) for stiffness. Max score: 10; 28.1 (CI, 24.0–32.2) for physical activity score: 85	N/A	N/A
Preoperative mental distress is associated with poorer physical improvements after revision total hip arthroplasty	Zhang et al. 2023 [25]	Revision THA	84	6 months + 2 years	64.75	One postoperative question about expectation fulfillment	6-point Likert scale for fulfillment of expectations	N/A	** > 6 months:** Distressed group (64.5%) & non-distressed group (94.1%); ** > 2 years:** Distressed group (63.6%) & non-distressed group (79.3%)	N/A

Furthermore, the methodological quality of the selected studies was assessed using the National Heart, Lung and Blood Institute (NIH) study quality assessment tool for observational cohort and cross-sectional studies [26]. Each study was judged on key concepts for internal validity, such as sample size, exposure/outcome measurement and compatibility of the groups. There were fourteen questions in total, for which studies could score a maximum of 14 points in sum. If there was any disagreement between the two reviewers, it was agreed that a discussion would be held to reach a point of consensus. This did not occur.

### Data analysis

Due to the heterogeneity of the measurement of patients’ expectations in the studies identified, it was not possible to statistically pool the data in a meta-analysis. Instead, a qualitative analysis was performed involving the summarization of study characteristics and outcomes, as well as a methodological assessment using the NIH quality assessment tool. Studies were noted as poor quality if they scored 0–4, fair if they scored 5–10 and good if they scored 11–14 out of 14 questions [27].

## Results

### Study selection process

**Fig. 1:**
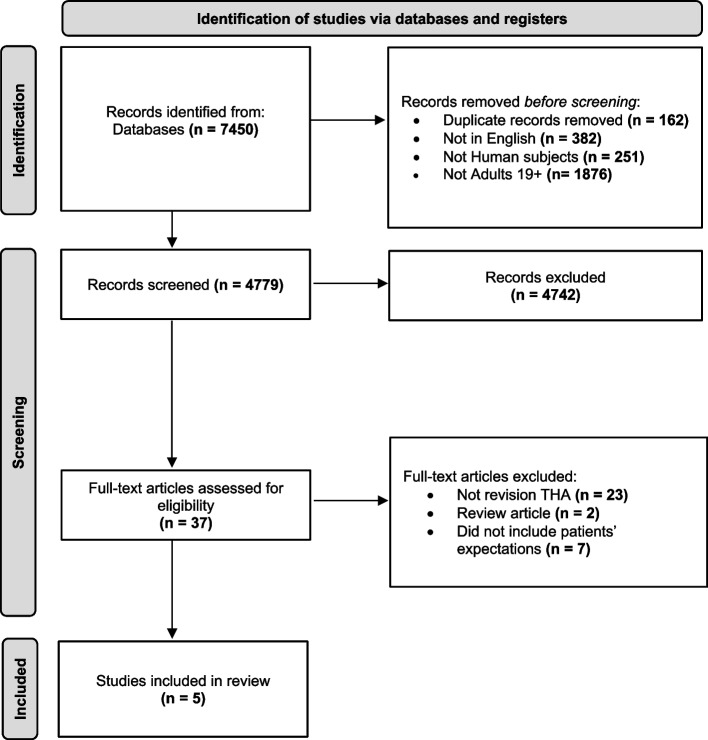
Flowchart of literature search and selection process

The literature search retrieved a total of 7,450 records. After removal of duplicates (*n* = 162), records not in English (*n* = 382), non-human studies (*n* = 251) and studies not on adults aged > 18 (*n* = 1,876), a total of 4,779 papers remained. After screening of the titles and abstracts, 4,742 studies were excluded, as they either did not assess patient expectations, did not include revision THA or were review articles. This left a total of 37 studies for further investigation. After full-text assessment, a further 32 articles were excluded, leaving 5 articles that met all the inclusion criteria [12, 14, 23–25] and were subsequently included in this review (Fig. 1).

### Study characteristics

Five cohort studies were included in this review. The sample size ranged from 60 to 320 participants. Four studies only included RTHA [12, 14, 23, 25] and one included both primary and RTHA [24]. In the assessment of expectations, two studies utilized a single item measurement which utilized either a three-point Likert-scale [23] or a six-point Likert-scale [25], two studies implemented a two-item instrument utilizing either a 4-point Likert scale [12] or a close-ended multiple-choice format [24]. One study modified the pre-existing Western Ontario and McMaster Universities Osteoarthritis Index (WOMAC scale—a validated instrument) to assess patients’ expectations of pain, stiffness and physical function in 6 months after the revision operation [14]. Overall, no validated instruments were used in the assessment of patients’ expectations in revision THA across all studies.

### Methodological quality

The average quality score was 9 out of 14 (range 7–9) (Table 2). As expected, the lowest scoring items were:

1. “Were the outcome assessors blinded to the exposure status of the participants?” —due to all studies having utilized a self-reported questionnaire;
2. “For exposures that can vary in amount or level, did the study examine different levels of the exposure as related to the outcome”;
3. “Was the exposure(s) assessed more than once over time?”—as the exposure was a single revision THA.

Other notable methodological shortcomings were the common lack of sample size justification and often absent statistical analyses of confounding variables.

### Expectations

The measurement of patient expectations varied across the studies included in this review. Two studies focused on revision longevity expectations [23, 24]. Barrack et al. implemented a single postoperative question concerning implant longevity and scaled responses using a 3-point Likert scale. Hellman et al. also measured implant longevity expectations using a single retrospective question and graded responses with close-ended multiple-choice questions.

One study prospectively measured the expectations of future pain and walking ability utilizing two questions scaled via a 4-point Likert scale [12]. One study assessed patients’ expectations of pain, stiffness and physical function utilizing the modified WOMAC scale [14]. These were measured prospectively and used a 5-point Likert scale. Two studies examined fulfillment of patients’ expectations after surgery [12, 25]. Eisler et al. postoperatively assessed fulfillment of expectations with two questions and utilized a 4-point Likert scale. Zhang et al. used one postoperative question with a 6-point Likert scale. Only one study measured how this in turn correlated with patient satisfaction [12].

### Pain

Patients’ expectations of pain were measured in two studies. Eisler et al. found that 92% of patients expected to have no pain or to have much less pain, and only 8% expected a slight reduction in pain. Haddad et al. reported an average score of 7.4/25 (CI 6.2–8.6) for pain, with a lower score conferring a low expectation of pain.

### Function

Function was assessed in two studies [12, 14]. Eisler et al. noted that 82% of patients expected the same walking ability as after the first THA or markedly improved walking ability, 15%, slightly improved and 3%, no difference in walking ability. Haddad et al. reported an average expectation of 28.1/85 (CI 24.0–32.2) for physical activity, with a lower score indicating a higher expectation of function. Additionally, only Haddad et al. assessed expectations on stiffness, with an average expectation of 3.5/10 (CI 3.0–4.0) for stiffness, with a lower score indicating a lower expectation for stiffness.

### Fulfilled expectations

Eisler et al. found that 55 and 69% of patients had fulfilled expectations regarding walking ability and pain. Furthermore, fulfilled expectations about pain and walking ability demonstrated a modest positive correlation with satisfaction (*r* = 0.46–0.47). The absence of complications was the only predictor of fulfilled pain expectations during the postoperative hospital period (odds ratio (OR) 4.8; 95% confidence interval (CI) 1.1–20.8). Zhang et al. found that at 6 months postoperatively, distressed patients had significantly lower rates of fulfilled expectations compared to non-distressed patients (64.5% vs. 94.1%, *P* = 0.027). At 2 years postoperatively, this was no longer significantly different (63.6% vs. 79.3%, *P* = 0.342).

### Implant longevity

Two studies assessed patients’ expectations concerning the longevity of their revision THA [23, 24]. Barrack et al. found that most patients, regardless of original implant longevity, expected their revision to last longer. In patients in whom the primary THA lasted < 5 years: 77% expected revision to last longer and in those where the primary lasted 5–10 years: 76% expected revision to last longer. If the primary lasted 10–15 years: 69% expected the revision to last longer and in those where the primary THA lasted > 15 years: 62% expected the revision to last longer. Hellman et al. found that 35% of patients expected the revision to last for the rest of their lives.

## Discussion

This review found that RTHA patients tend to have unrealistically high expectations regarding pain relief, improvement in movement, and implant longevity. Furthermore, distressed patients are less likely to have their expectations fulfilled postoperatively in the short term [25]. Given poorer outcomes with revision surgery versus primary THA, these expectations are unlikely to be fulfilled and may result in patient dissatisfaction [8, 12, 14]. Only one study [12] assessed how fulfillment of these expectations correlated with postoperative satisfaction, revealing a moderate positive correlation with expectations of pain and walking ability. However, overall, there is a paucity of research concerning expectations following RTHA procedures, despite the higher risk of complications [28]. Additionally, there is significant variability in the way expectations are measured.

Important areas that need to be addressed in future research include (1) The theoretical framework of expectations; (2) the measurement of expectations; (3) the correlation of psychological and other demographic factors and (4) the relationship between fulfilled expectations and satisfaction.

Firstly, none of the papers in this review provided a definition of patient expectations. The absence of a consistent theoretical framework for expectations lends itself to an increased propensity for the heterogeneous use of terminology and measurements. If left unaddressed, this can lead to research plagued by discontinuity and poor methodological quality. In the past, several reviews [29–31] have acknowledged patient expectations as being a complex multifaceted construct. Kravitz [31] made a distinct delineation between value (reflecting the patient’s wishes/hopes) and probability expectations (the likelihood that an event will occur). Furthermore, Bandura [32] separated efficacy from outcome expectations. Given the different perspectives on expectations, it is necessary to utilize a consistent framework to allow for accurate classification and subsequent assessment. For example, Hobbs et al. [33] successfully utilized the International Classification of Functioning, Disability and Health (ICF) framework to classify patients’ expectations in primary THA. This involved assigning patients’ expectations to one of three domains: activity limitations, impairments to bodily function and structure, and participation restrictions. It was found that patients generally focused more on the recovery of valued activities rather than the reversal of their functional impairment. In future RTHA research investigating patient expectations, researchers should aim to map their findings to each of the core ICF constructs. If performed consistently, this has the potential to lead to more uniformity of definitions, better integration of data amongst different studies and improved validation of measurement instruments. Additionally, this method could be used to ascertain whether certain expectation domains, e.g., impairment, activity limitations or participation restriction expectations are predictors of patient reported outcome measures (PROMs).

As mentioned previously, the lack of a consistent theoretical framework for patient expectations has likely contributed to the absence of a valid and standardized measurement tool. This prevents the effective integration and comparison of data across studies [22]. Each study in this review implemented a unique instrument that was only used for one investigation. They often lacked a rationale behind their development, or data on reliability and validity, which limits the credibility of evidence collected. This issue has affected both primary THA research and research in other fields such as psychotherapy, where, for example, Constantino et al. [34] reported that the majority (67%) of measurements were of poor quality. A possible strategy may be to either adapt an already well-established patient-reported outcome tool (such as the WOMAC) or use a theory-guided approach, with testing in independent samples to gather data on reliability, construct validity and predictive validity. Alternatively, the Hospital for Special Surgery Total Hip Replacement Expectations Survey (HSS-HRES) could be used for RTHA patients. This survey is a well-validated 18-question expectations survey that is graded on a 5-point Likert scale and has been used effectively in past THA research [35]. Regardless, future researchers should aim to use a validated instrument.

Additionally, half of the studies included in this review measured patients’ expectations in the postoperative period. This is not optimal and increases the risk of bias, as the patients may not be able to accurately recall their preoperative expectations due to the time elapsed [36]. Furthermore, since patient dissatisfaction is secondary to a disequilibrium between expectations and fulfilled expectations [37], patients may therefore alter their expectations to match their current status, to prevent dissatisfaction [38]. A Canadian study in 2006, reported this phenomenon regarding total knee arthroplasty, where 35% of patients over- or underestimated their preoperative level of functioning [39]. However, there is another issue purported by Haanstra et al. which pertains to the timing of expectation measurement [22]. Given that patients’ expectations are likely to be widely influenced by their doctor, it is possible that the longer the patient is in contact with them and the later their expectations are measured, the more realistic and reliable they may be. Currently no investigation has measured the influence of time of measurement, but it is a variable to keep in mind, which could be offset by collecting data at different time points.

Moreover, only one study in this review collected data in the pre- and postoperative period to assess the percentage of fulfilled expectations, and only this study analyzed the correlation between fulfilled expectations and satisfaction [12]. Whilst expectations are an important preoperative factor, it is the fulfillment of these expectations that has been shown to be the more significant determinant of patient-reported outcomes and satisfaction [40]. High expectations are not inherently detrimental, but unrealistic expectations are [40]. Therefore, it is important to assess the percentage of patients with fulfilled expectations, as this information can be used to foster realistic, high expectations through effective preoperative education.

If patients are to be measured in the postoperative period, the length of the follow-up period needs to be addressed, as it may influence findings. Barlow et al. found that expectations may take up to two years post-surgery before they are fulfilled, due to function having the potential to improve for up to two years, alluding to the existence of a timing bias [41].

Finally, half of the available literature did not include a multivariate analysis of confounding variables such as age, gender, ethnicity and preoperative education level despite their influence on patient expectations [35, 42]. Furthermore, psychological factors (depression, optimism and catastrophizing), which may interact with expectations or treatment outcomes, were rarely analyzed [22]. Future research should try to delineate these factors for further consideration.

A promising area of focus for future research is the consenting process. Patient recall of the consenting process, and the relevant risks and outcomes, is frequently poor [43]. A recent study demonstrated that patients undergoing THA, who were consented with the generic consent form, only recalled 0.67 risks four weeks after surgery. In contrast, those who were given a surgery-specific consent form, recalled 1.43 risks on average [44]. This surgery-specific consent form listed potential adverse events alongside appropriate explanations. With regards to RTHA, this could be implemented with the addition of a section on postoperative outcomes. This would help to ensure that patients have a better comprehension of the procedure and retain more information. This may, therefore, lead to more realistic expectations that can be fulfilled.

This study has limitations that need to be considered. Firstly, a meta-analysis was not possible due to the heterogeneity in the papers included and the poor standard of reporting. And so, we performed a qualitative analysis. However, a thorough, definitive analysis of the data is not possible using this method. Secondly, only a limited number of studies were available for review, due to the lack of research in this area. As a result, there are limited data available to analyse, which may not fully represent patient expectations. The data were also relatively old, with only 2 references being < 10 years ago. Patient expectations may have improved since then with changes in perioperative information. Therefore, the strength of conclusions made in the paper may not be accurate and should be taken with caution. Although a limitation, this highlights a clear deficit in current research that needs to be addressed.

As conclusions from RTHA literature are limited, we can look at adjacent literature concerning total knee arthroplasty (TKA), to better understand what patients tend to expect with a joint replacement procedure. Similarly, TKA patients have been shown to have unrealistically high expectations regarding postoperative pain, function and recovery [45]. Moreover, patient satisfaction has been shown to be highly correlated with expectation fulfillment [45]. Recent research has demonstrated improvements in patients’ WOMAC pain and satisfaction scores at over 1 year post operation in TKA patients, by setting realistic expectations [46]. Although a different procedure/patient demographic, these findings are similar to the current evidence base for RTHA and reinforce the importance of setting appropriate baseline patient expectations through perioperative counselling, to foster better PROMs.

## Conclusion

A definitive conclusion is limited by the sparse data available. However, the current literature demonstrates that revision THA patients tend to have unrealistic expectations with regards to pain relief, function and implant longevity. Realistic patient education prior to surgery is necessary to avoid expectation/outcome mismatch and hence dissatisfaction. Nevertheless, this review demonstrates the lack of adequate research on patients’ expectations in revision THA, both in terms of absolute numbers, and methodological quality. More research is needed, which utilizes a standardized approach in assessment, in order to foster a better understanding of the relationship between patient expectations and postoperative outcome measures. Only then, can this information be effectively applied clinically to improve the outcome of revision THAs. We suggest counselling of patients before surgery and using a procedure-specific consent. As to collection of pre- and postoperative data—postoperative data should be collected at different points of time as the patients’ outcomes improve with time and so will the outcome and expectations. Patients-reported outcomes are a better tool to assess the patient outcomes.

## Data Availability

Not applicable.

## Abbreviations

THA: Total Hip Arthroplasty
RTHA: Revision Total Hip Arthroplasty
NIH: National Heart, Lung and Blood Institute
WOMAC: Western Ontario and McMaster Universities Arthritis Index
PROM: Patient reported outcome measure
